# Artepillin C, a Major Ingredient of Brazilian Propolis, Induces a Pungent Taste by Activating TRPA1 Channels

**DOI:** 10.1371/journal.pone.0048072

**Published:** 2012-11-02

**Authors:** Taketoshi Hata, Shigemi Tazawa, Shozo Ohta, Mee-Ra Rhyu, Takumi Misaka, Kenji Ichihara

**Affiliations:** 1 Nagaragawa Research Center, API Co., Ltd., Nagara, Gifu, Japan; 2 Functional Food Technology Research Group, Korea Food Research Institute, Bundang-gu, Sungnam-si, Gyeonggi-do, Republic of Korea; 3 Department of Applied Biological Chemistry, Graduate School of Agricultural and Life Sciences, The University of Tokyo, Bunkyo-ku, Tokyo, Japan; Duke University, United States of America

## Abstract

Brazilian green propolis is a popular health supplement because of its various biological properties. The ethanol extract of Brazilian green propolis (EEBP) is characteristic for its herb-like smell and unique pungent taste. However, the ingredients responsible for its pungency have not yet been identified. This study provides the first evidence that artepillin C is the main pungent ingredient in EEBP and that it potently activates human transient receptor potential ankyrin 1 (TRPA1) channels. EEBP was fractionated using column chromatography with a step gradient elution of an ethanol-water solution, and the fractions having the pungent taste were determined by sensory tests. HPLC analysis revealed that the pungent fraction was composed primarily of artepillin C, a prenylated derivative of cinnamic acid. Artepillin C was also identified as the pungent compound of EEBP by organoleptic examiners. Furthermore, the effects of artepillin C and other cinnamic acids found in EEBP on TRPA1 channels were examined by calcium imaging and plate reader-based assays in human TRPA1-expressing cells to investigate the molecular mechanisms underlying their pungent tastes. Artepillin C and baccharin activated the TRPA1 channel strongly, whereas drupanin caused a slight activation and *p*-coumaric acid showed no activation. Because the EC_50_ values of artepillin C, baccharin, and allyl isothiocyanate were 1.8 µM, 15.5 µM, and 6.2 µM, respectively, artepillin C was more potent than the typical TRPA1 agonist allyl isothiocyanate. These findings strongly indicate that artepillin C is the main pungent ingredient in EEBP and stimulates a pungent taste by activating TRPA1 channels.

## Introduction

**Figure 1:**
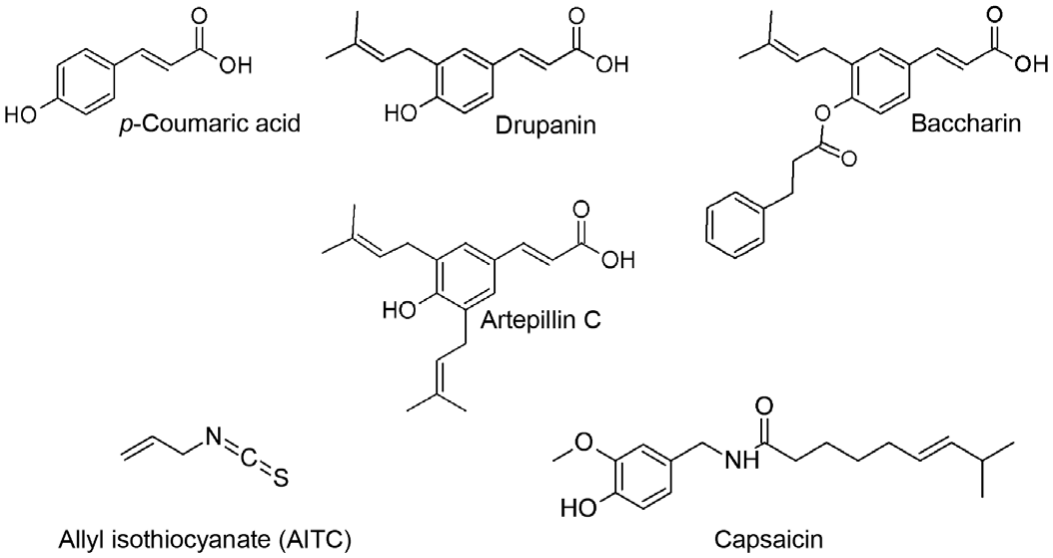
Chemical structures of *p*-coumaric acid, drupanin, artepillin C, baccharin, allyl isothiocyanate (AITC), and capsaicin.

Propolis is a plant mastic produced by honeybees from resinous substances collected from tree buds and sap. Bees bring the resin to the hive and mix it with their own secretions, such as bee wax and saliva. Propolis is thought to be used to seal unnecessary holes and cracks in the hive and to protect the bee colony against intruders, viruses, fungi, and bacteria [1], [2]. Propolis is used in folk medicine in many regions worldwide and has been reported to have various biological and physiological activities, such as antibacterial [3], [4], antiviral [5], anti-inflammatory [6], and anticancer [7], [8], [9], [10] effects. Propolis contains numerous chemical ingredients, including cinnamic acid derivatives (e.g., *p*-coumaric acid, drupanin, artepillin C, and baccharin (Fig. 1)), benzoic acids, substituted phenolic acids, flavonoids, and amino acids [11], [12], [13], and the chemical composition of propolis depends on various factors, such as the geographical origin, types of plant sources [5], [13], and season of the year. *Baccharis dracunculifolia* DC (Asteraceae) [14], [15], [16], a plant native to Brazil, is the most important botanical source of southeastern Brazilian propolis, which is known as green propolis because of its deep green color.

In Japan, the ethanol extract of Brazilian green propolis (EEBP) is used as a dietary supplement and an ingredient in health products. EEBP has an herb-like smell and a unique bitter and pungent taste. The pungent taste is probably related to the palatability of propolis. However, there has been no study regarding the pungent taste of propolis. Accordingly, we aimed to identify the pungent compounds in EEBP in the present study.

Transient receptor potential ankyrin 1 (TRPA1), formerly known as ANKTM1, is a non-selective Ca^2+^-permeable cation channel that is co-expressed with transient receptor potential vanilloid 1 (TRPV1) in nociceptive neurons [17]. TRPA1 may play an important role in the transduction of low-temperature and inflammatory stimulation into electric nerve signals [18]. Furthermore, several reports indicate that TRPA1 is activated by various food components, such as mustard oil [19], wasabi (allyl isothiocyanate, a spice specific to Japan) [19], garlic extract (allicin) [20], and cinnamon oil (cinnamaldehyde) [21].

Thus, we investigated whether the pungent compounds identified in EEBP would activate TRPA1 on the following basis: the pungency of EEBP differs from that of hot peppers, in which active capsaicin is a typical agonist of TRPV1; and EEBP contains cinnamic acid derivatives structurally similar to cinnamaldehyde, a typical agonist of TRPA1. In the present study, we used calcium influx imaging in hTRPA1- and hTRPV1-expressing cells to examine the stimulating effects of EEBP ingredients on these TRP channels.

## Results

### Identification of the Pungent Ingredient in EEBP

**Figure 2:**
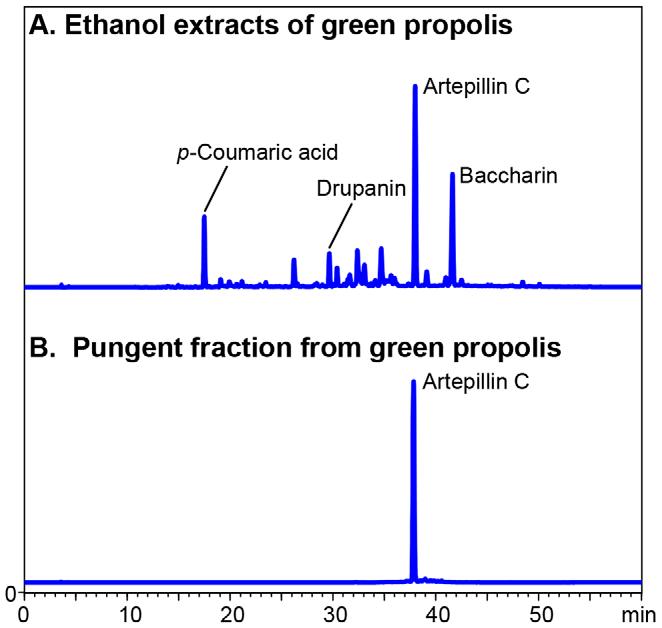
HPLC chromatograms of the EEBP and the fraction containing the pungent ingredient. (A) The following major compounds were identified in the ethanol extract of Brazilian green propolis (EEBP) : *p*-coumaric acid, drupanin, artepillin C, and baccharin. (B) One peak was found at the same retention time as artepillin C in the fraction with the most pungent taste.

We fractionated EEBP into 18 fractions and tasted them to identify the pungent fraction. As a result, a noticeable pungent taste was found in the 50–60% ethanol-eluted fractions. Further purification of the fractions was performed using column chromatography, and the purified fraction was subsequently analyzed by HPLC. The HPLC results showed that artepillin C accounted for more than 90% of the solid contents of the pungent fraction (Fig. 2). Thus, we performed an organoleptic evaluation of artepillin C in water and soft drink solutions. All of the examiners felt irritating pungency at the pharynx rather than in the mouth a few seconds later after administration. Regarding the water solution, fewer than half the examiners judged the 0.1 mg/ml artepillin C solution to be slightly pungent (Table 1), and all of them rated obvious pungency at 0.225 mg/ml artepillin C. Similarly, the 0.3 mg/ml soft drink solution of artepillin C was judged to be obviously pungent.

**Table 1:** Pungency threshold of artepillin C by organoleptic examination.

Artepillin C (mg/ml)	In water	In drink formulation
0.300	Obviously	Obviously
0.225	Obviously	Slightly
0.150	Slightly	Slightly
0.100	Slightly	Slightly
0.075	Not pungent	Not pungent
0.030	Not pungent	Not pungent

### Responses of Cells Stably Expressing Human TRPA1 to Propolis Ingredients

**Figure 3:**
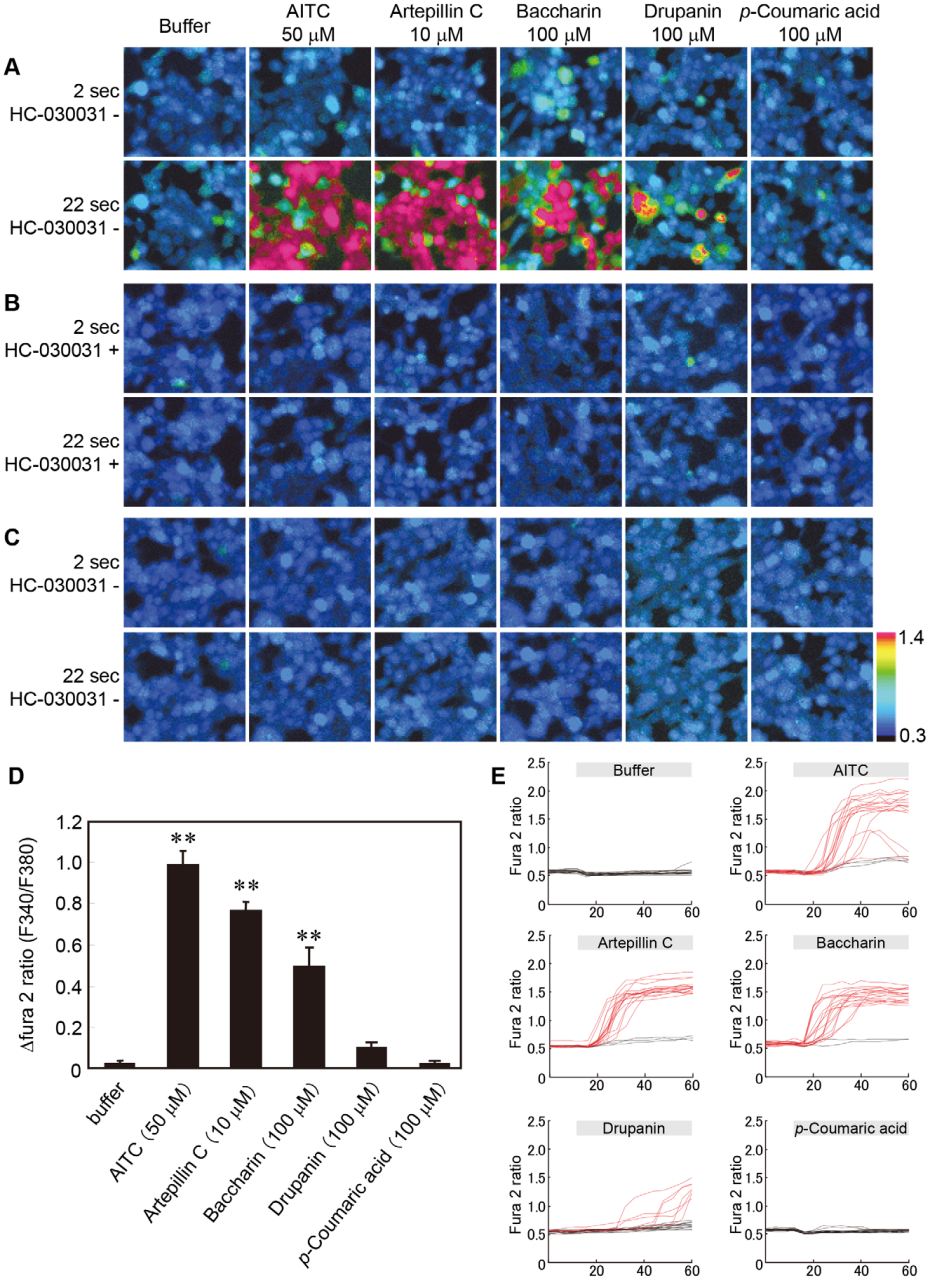
The responses of human TRPA1-expressing cells to the application of the compounds in EEBP. (A–C) The compounds identified in the HPLC chromatogram were applied to hTRPA1-expressing (A and B) and Flp-In 293 cells (C). Representative ratiometric images of the cells obtained by the Ca^2+^ imaging analysis are shown in each panel. The upper and lower columns indicate the images obtained at approximately 2 and 22 sec after the stimulation, respectively. The applied concentrations are indicated in the upper part of the panels. The responses were recorded in the absence (A and C) or presence (B) of 50 µM HC-030031, a TRPA1-specific inhibitor. (D) The responses to the test solutions were analyzed in 100 randomly selected cells. The values represent the means ± S.E.M. (n = 4). ** indicates *p*<0.01 vs. buffer. (E) Sequential F340/F380 ratiometric values were measured for 20 randomly selected cells. The red line indicates that the value changed by more than 0.3.

To verify the response of hTRPA1-expressing cells, calcium imaging analysis was conducted using artepillin C and other cinnamic acid derivatives found in EEBP, including baccharin, drupanin and *p*-coumaric acid (Fig. 1). Intense responses were observed upon treatment with artepillin C, baccharin, and AITC (positive control) at concentrations of 10 µM, 100 µM, and 50 µM, respectively, and slight responses were observed with drupanin (100 µM); in contrast, *p*-coumaric acid (100 µM ) induced a low response of hTRPA1 (Fig. 3A). The hTRPA1-activated Ca^2+^ influx in response to these compounds was completely inhibited in the presence of HC-030031 (50 µM), a TRPA1 antagonist (Fig. 3B). In addition, when Flp-In 293 cells were used to examine the dependence on hTRPA1 expression, no calcium ion influx was found at the corresponding concentrations of the compounds, including AITC (Fig. 3C).

**Figure 4:**
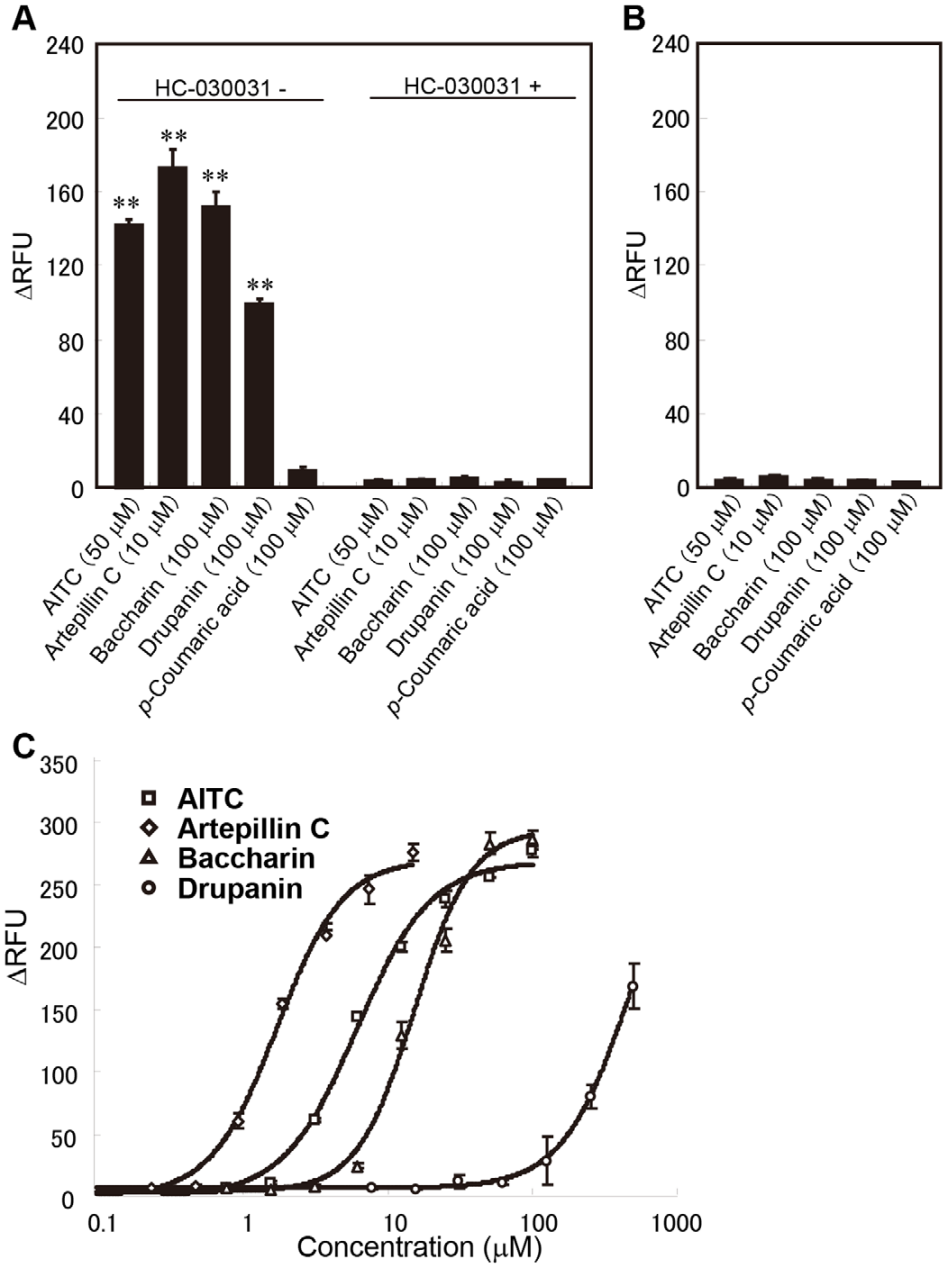
Effects of artepillin C, baccharin, drupanin, and AITC on the responses of hTRPA1-expressing cells. The cellular responses to the test compounds were examined using a plate reader-based assay in the presence or absence of 50 µM HC-030031, a TRPA1-specific inhibitor, in hTPRA1-expressing cells (A) or Flp-in 293 cells (B). Each column represents the means ± S.E.M. (n = 4). ** indicates *p*<0.01 for each substance (comparison between the absence and presence of HC-030031) (Tukey’s multiple comparison test; the equality of variances was tested using the Levene test). (C) Dose-response curves for hTRPA1 activation. The values represent the means ± S.E.M. (n = 3).

The response of the hTRPA1-expressing cells was also confirmed using a plate reader-based assay. Artepillin C, baccharin, and drupanin resulted in significant differences in the fluorescence intensity compared with that of the buffer alone whereas *p*-coumaric acid did not (Fig. 4A). These positive responses disappeared in the presence of HC-030031 (50 µM, Fig. 4A).

### Dose-response Curves and EC_50_ Values for hTRPA1 Activation by Propolis Ingredients

Dose-response curves were generated for the responses to artepillin C, baccharin, drupanin, and AITC to determine the intensity of hTRPA1 activation. All of the compounds tested activated hTRPA1 in a concentration-dependent manner (Fig. 4C), with artepillin C activating hTRPA1 at the lowest concentration among the compounds tested (Fig. 4C). The EC_50_ values for artepillin C, baccharin, and AITC were 1.8 µM, 15.5 µM, and 6.2 µM, respectively, whereas the dose-response curve of drupanin did not reach a plateau at concentrations up to 500 µM. The EC_50_ value of drupanin, therefore, was estimated to be at least 250 µM.

### Measurement of the Responses of hTRPV1-expressing Cells to Propolis Ingredients

**Figure 5:**
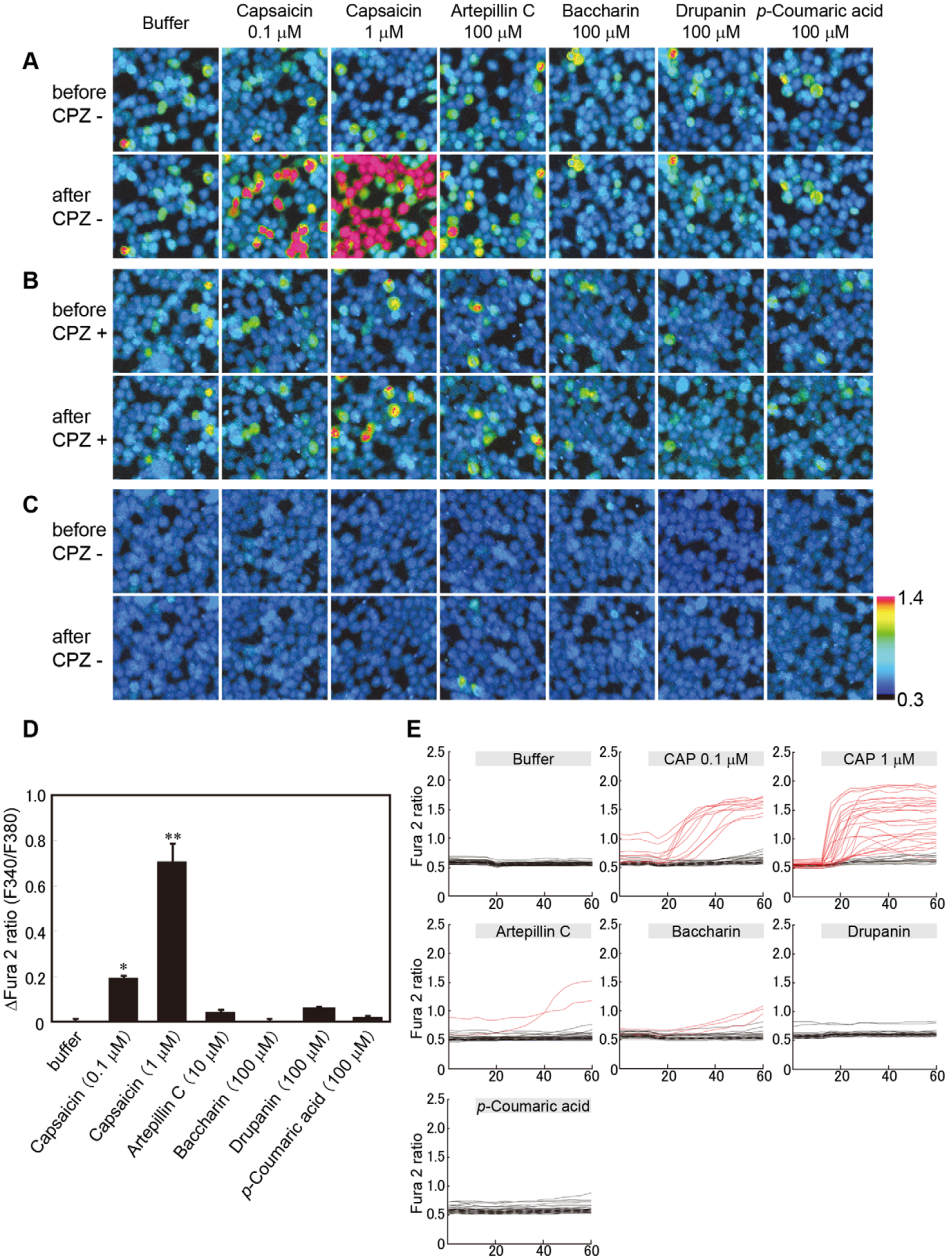
Effects of artepillin C, baccharin, drupanin, and capsaicin on the responses of hTRPV1-expressing cells. (A–C) Representative ratiometric images of Fura 2-loaded hTRPV1-expressing (A and B) and mock-transfected (C) HEK293T cells. The upper and lower columns indicate the images obtained before and after stimulation, respectively. The applied concentrations are indicated in the upper part of the panels. The responses were recorded in the absence (A and C) or presence (B) of 30 µM capsazepine, a TRPV1-specific inhibitor. (D) The responses to the test solutions were analyzed in 100 randomly selected cells. The values represent the means ± S.E.M. (n = 4). * or ** indicates *p*<0.05 or 0.01 vs. buffer. (E) Sequential F340/F380 ratiometric values were measured for 40 random selected cells. The red line indicates that the value changed by more than 0.3.

**Figure 6:**
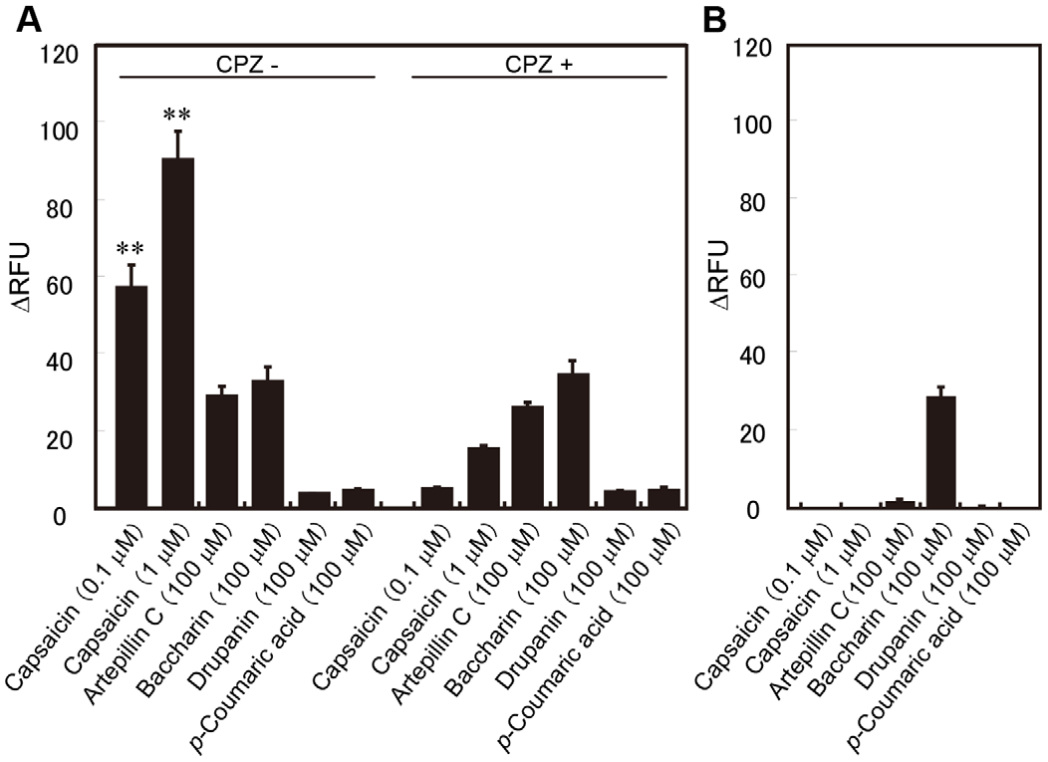
Effects of artepillin C, baccharin, drupanin, and capsaicin on the responses of hTRPV1-expressing cells. The cellular responses to the test compounds were examined using a plate reader-based assay in the presence or absence of 30 µM capsazepine in hTRPV1-expressing (A) or mock-transfected (B) HEK293T cells. Each column represents the means ± S.E.M. (n = 3). ** indicates *p*<0.01 for each substance (comparison between the absence and presence of capsazepine) (Dunnett’s multiple comparison test; the equality of the variances was tested using the Bartlett’s test).

Imaging analysis and a plate reader-based assay were conducted to confirm the response of the hTRPV1-expressing cells. In imaging analysis, artepillin C and baccharin slightly increased the Ca^2+^ influx at concentrations higher than 100 µM (Fig. 5A and E), and their responses were not inhibited by the hTRPV1 antagonist capsazepine (30 µM) (Fig. 6). Moreover, neither compound could be assayed at concentrations higher than 100 µM because of their insolubility. Thus, it was judged that artepillin C and baccharin did not increase the Ca^2+^ influx by stimulating hTRPV1 channels, but the induced non-specific responses resulted in a slight intracellular Ca^2+^ mobilization. Thus, neither dose-response curves of artepillin C and baccharin for the hTRPV1 activation nor EC_50_ values could be obtained. In contrast, capsaicin (0.1 µM) markedly increased the Ca^2+^ influx in the hTRPV1-expressing cells, and this influx was completely blocked by capsazepine (30 µM). These results indicated that artepillin C only had a slight effect on hTRPV1 activation at 100 µM.

## Discussion

Many studies have been conducted to elucidate the health benefits and the mechanisms of propolis, including studies on its effects on bacteria [3], [4], viruses [5], inflammation [6], and cancer [7], [8], [9], [10]. However, little has been reported on the unique pungent taste of propolis, which actually affects its palatability and consumer acceptability. The present study demonstrated that artepillin C primarily contributed to the pungent taste of EEBP (Fig. 2). Furthermore, we found that artepillin C strongly stimulates hTRPA1 channels, which are activated by pungent foods, such as wasabi (its main pungent ingredient is AITC), a typical pungent food in Japan [19]. The present results also indicate that the pungent taste of propolis can be considered a good marker with respect to both its functional quality and effectiveness because artepillin C comprises approximately 14% of EEBP solids [22] and has been reported to have anti-oxidative [23], anti-diabetes [24], and anti-tumor [25], [26] effects.

In contrast to EEBP, the ethanol extract of European propolis, a poplar type of propolis, has no pungent taste (data not shown). We also found that EEBP contains cinnamic acid derivatives (Fig. 2), whereas other types of propolis do not (data not shown). We therefore analyzed the cinnamic acids using HPLC with UV absorbance at 280 nm followed by an organoleptic examination of the candidate compounds, which showed that artepillin C was responsible for the pungent taste (Table 1).

In this paper, we evaluated the effects of cinnamic acid derivatives on hTRPA1 ion channels, which are activated by pungent compounds, to confirm the results of the organoleptic test (Figs. 3 and 4). In accordance with the result of the organoleptic assessment, artepillin C activated hTRPA1 channels, and this effect was approximately three times more potent than the typical hTRPA1 agonist AITC (Fig. 4). Because the effect of artepillin C on hTRPA1 was completely inhibited by the TRPA1 antagonist HC-030031 (Figs. 3 and 4A) and artepillin C did not cause Ca^2+^ influx in non-hTRPA1-expressing Flp-In 293 cells (Fig. 3C), artepillin C is an agonist for hTRPA1 channels. In contrast, the activating effects of the other cinnamic acids (baccharin and drupanin) were considerably less than artepillin C, and *p*-coumaric acid did not activate the hTRPA1 channels. These findings also support the results of the organoleptic test for pungency.

The TRPV1 channel is activated by capsaicin, a pungent compound in hot peppers [27]. Although the pungent taste of EEBP differs from that of hot peppers, the effect of artepillin C on hTRPV1 channels was examined in this study (Fig. 5). As expected, artepillin C had a limited effect on the hTRPV1 channel, even at high concentrations up to 100 µM (Fig. 5). This result also provided evidence that the pungent taste of EEBP was primarily mediated through the activation of hTRPA1 by artepillin C.

TRPA1 is a six-pass transmembrane protein, and its active site is located in the cytoplasmic region [28], [29]. Recent studies on the mechanism of TRPA1 activation indicate that due to the membrane permeability and the electrophilicity of ligands such as AITC and cinnamaldehyde, these ligands directly form adducts with specific cysteine residues in the cytoplasmic N-terminus of the TRPA1 channel [28], [29]. The α, β-unsaturated carbonyl groups of the ligands result in Michael additions to the SH groups of the cysteine residues, resulting in the activation of the TRPA1 channel. Because of its high hydrophobicity, artepillin C is thought to easily pass through cell membranes [30]; thus, it is likely that artepillin C has a similar mechanism to other ligands. The other cinnamic acid derivatives also have α, β-unsaturated carbonyl groups in their chemical structures, and baccharin seem to be a more hydrophobic ligand than artepillin C because it had a longer retention time during the reversed-phase HPLC analysis (see Fig. 2). These findings suggest that artepillin C has a particular structure for favorably interacting with the ligand-catalytic site of the receptor channel and that the abilities of drupanin and *p*-coumaric acid to activate hTRPA1 channels might depend on their hydrophobicity (refer to the partition coefficient values [30]). Recently, Gachons et al. reported that oleocanthal, which is found in extra-virgin oil, has an unusual pungency and activates TRPA1 via a mechanism that is not related to the covalent cysteine modification [31]. Interestingly, they also reported that the pungent sensation elicited by oleocanthal occurred exclusively in the throat and not at the tongue. Because examiners felt the pungent sensation caused by artepillin C at the pharynx in the organoleptic test, artepillin C may activate the TRPA1 channels located at the pharynx via the same mechanism as oleocanthal. However, the exact reasons for the observed differences between the compounds in hTRPA1 activation are unclear.

We conclude that artepillin C, which was a potent agonist of TRPA1, is the main pungent ingredient of Brazilian green propolis. TRPA1 is present in sensory neurons and is mainly expressed on the afferent endings in mucosal and mesenteric mechanoreceptors [32]. Nozawa et al. reported that human TRPA1 channels are abundantly expressed in gastrointestinal tissues, in addition to their expression in visceral afferents, and indicated that hTRPA1 channels are located in enterochromaffin cells and are involved in gastrointestinal functions through serotonin release [32]. In addition, Penuelas et al. reported that TRPA1 agonists contract the mouse intestine [33]. Thus, the present findings may lead to a possibility that the ethanol extract of Brazilian green propolis has a novel functional effect on the gastrointestinal tract of humans.

## Materials and Methods

### Materials and Chemicals

We used Brazilian green propolis produced in the state of Minas Gerais. The main plant source is *B. dracunculifolia* DC [14], [15], [16]. A lump of propolis was crushed, soaked in 95% ethanol and stirred for 24 h at room temperature; the propolis grounds were then filtered out. The filtrate was stored at −20°C for more than 24 hours, and the insoluble matter was removed by filtration. The ethanol-extracted solution was prepared by adjusting its solid content to 55% (w/w); this mixture was designated as EEP-B55 (API Co., Ltd., Gifu, Japan).

Artepillin C (3,5-diprenyl-4-hydroxycinnamic acid) was extracted from EEP-B55 and purified in our laboratory. Baccharin [(*E*)-3-prenyl-4-(2,3-dihydrocinnamoyloxy) cinnamic acid] was synthesized by Konan Chemical Industry Co., Ltd. (Osaka, Japan). Drupanin [(*E*)-3-prenyl-4-hydroxycinnnamic acid] was synthesized by Dr. Uto (Tokushima University, Japan). *p*-Coumaric acid and allyl isothiocyanate (AITC) were purchased from Tokyo Chemical Industry Co., Ltd. (Tokyo, Japan). HC-030031 was purchased from Sigma Aldrich (St. Louis, MO, USA). Capsaicin (purity >60%) was purchased from Wako Pure Chemical Industries, Ltd. (Osaka, Japan). Capsazepine was purchased from Biomol International (Plymouth Meeting, PA, USA).

### Fractionation

Ten grams of EEP-B55 was applied to a Chromatorex ODS DM1020T (Fuji Silysia Chemical Co., Ltd., Aichi, Japan) column (Φ50 mm×250 mm) and eluted with a step gradient of ethanol-water solutions (30, 40, 50, 60, 70, 80, 90, and 100% (v/v)). Each fraction was dried and reconstituted with 5 ml of ethanol for pungency tasting. The pungent fraction was further purified by repeated chromatography with the same ODS column (Φ32 mm×250 mm) and a step gradient elution of ethanol-water solutions (50 and 60% (v/v)). The composition of the test fractions was subsequently analyzed using high-performance liquid chromatography (HPLC).

### HPLC Analysis

The solid contents of the test fractions were measured after vacuum drying, and the final solid concentration was adjusted to 2 mg/ml with methanol. These samples were subjected to HPLC analysis using the Prominence high-performance liquid chromatograph system (Shimadzu, Kyoto, Japan) with UV absorbance detection at 280 nm. The chromatography was performed at 40°C using a Shim-Pak CLC-ODS column (6.0 mm ID×150 mm L; Shimadzu, Kyoto, Japan) and gradient elution mode under the following conditions: solvent A was HPLC-grade water containing 2% acetic acid, and solvent B was acetonitrile containing 2% acetic acid; the gradient of solvent A and B was 0–100% B for 50 min and 100% B for 10 min, and the flow rate was 1.0 ml/min.

### Organoleptic Examination

An organoleptic examination was performed by seven independent examiners (healthy adults of both sexes whose ages ranged between 20 and 60 years) to determine the pungency threshold of the test compound. The test compound was dissolved in 1 ml of ethanol and diluted to a volume of 50 ml with purified water or a soft drink solution (drink solution). The recipe for the drink solution is as follows: 0.1 g/ml of high-fructose corn syrup (Miekaryo Co., Ltd., Mie, Japan), 0.08 g/ml of isomaltooligosaccharide (B Food Science Co., Ltd., Aichi, Japan), and 0.005 mg/ml of citric acid (Archer Daniel Midland Company, IL, USA). The final concentrations of the test compound were 0.3 mg/ml, 0.225 mg/ml, 0.15 mg/ml, 0.1 mg/ml, 0.075 mg/ml, and 0.03 mg/ml. Using a pipette, each of the test solutions (2.0 ml) was applied to the entire tongue surface of the examiners in ascending order of concentration. The examiners judged the test solutions as “not pungent”, “slightly pungent”, or “obviously pungent”.

### Cell Culture and Transfection

The entire coding region for human TRPA1 (NCBI refseq number: NM_007332.2) and human TRPV1 (NCBI refseq number: NG_029716.1) was amplified by PCR using commercially available plasmids (Open Bio Systems), and the products were subcloned into the pcDNA5/FRT vector (Invitrogen, Carlsbad, CA, USA) and pEAK10 vector (Edge Biosystems, Gaithersburg, MD, USA), respectively. The sequences were confirmed with sequencing using an ABI 3130 DNA genetic analyzer (Applied Biosystems, Foster City, CA, USA).

The Flp-In system (Invitrogen) was used to construct the hTRPA1-expressing cell line. Flp-In 293 cells (Invitrogen) were cultured at 37°C in Dulbecco’s modified Eagle’s medium (Sigma-Aldrich Japan, Tokyo, Japan) supplemented with 10% fetal bovine serum (Invitrogen). The cell lines were generated according to the manufacturer’s protocol for the Flp-In pcDNA5/FRT Complete system (Invitrogen). In brief, the Flp-In 293 cells were transfected with both the constructed hTRPA1-expressing plasmid and pOG44 (Invitrogen) using Lipofectamine 2000 (Invitrogen). Forty-eight hours after transfection, the hTRPA1 expressing-cells were selected by treatment with 100 µg/ml hygromycin B (Invitrogen) for 2–3 weeks. Antibiotic-resistant cells were selected, cultured, and used for measuring cellular responses to the pungent components.

The hTRPV1-expressing cells were prepared by transient transfection. HEK293T cells were cultured at 37°C in Dulbecco’s modified Eagle’s medium supplemented with 10% fetal bovine serum. The cells were seeded in 6-well plates and transiently transfected with the hTRPV1 expression plasmid using the Lipofectamine 2000 reagent. The cellular responses were measured 24–28 hrs after transfection.

### Test Solutions for Calcium Imaging Analysis and Plate Reader-based Assay

The test compounds were dissolved in dimethyl sulfoxide and diluted with the assay buffer to the desired concentration. The calcium-containing buffer (assay buffer) included 10 mM 4-(2-hydroxyethyl)-1-piperazeneethanesulfonic acid (HEPES), 130 mM NaCl, 1 mM glucose, 5 mM KCl, 2 mM CaCl_2_, and 1.2 mM MgCl_2_ (pH adjusted to 7.4 with NaOH).

### Measurement of the Responses of hTRPA1- or hTRPV1-expressing Cells by Calcium Imaging Analysis

On the day before the analysis, the cells were trypsinized and seeded into 96-well Lumox multiwell black-wall plates (SARSTEDT AG & Co., Nümbrecht, Germany). After 18–26 hrs, the cells were washed with the assay buffer and then loaded with 5 µM Fura 2-AM (Invitrogen) in 100 µl of the assay buffer for 30 min at 27°C. The cells were washed again with the assay buffer and incubated for up to 15 min at room temperature. The cells were stimulated with the test compound by adding 100 µl of the test solution.

The intensities of Fura 2-AM fluorescence emissions resulting from the excitation at 340 nm and 380 nm were measured at 510 nm using a computer-controlled filter exchanger (Lambda 10-3; Sutter, San Rafael, CA, USA), a CoolSNAP HQ2 charge-coupled device camera (Photometrics, Tucson, AZ, USA), and an inverted fluorescence microscope (IX-71; Olympus, Tokyo, Japan). The images were recorded at 4-sec intervals and analyzed using MetalFluor software (Molecular Devices, Sunnyvale, CA, USA). The changes in the intracellular calcium ion concentrations were estimated from the changes in the ratio of the fluorescence intensity at the two excitation wavelengths (*F*_340_/*F*_380_). The TRPA1-specific inhibitor HC-030031 was used to demonstrate channel specificity for hTRPA1 at a concentration of 50 µM, and the hTRPV1 antagonist capsazepine (a synthetic analog of capsaicin) was also used at a concentration of 30 µM. The inhibitor was mixed in the test solution before the experiment. To perform the statistical analysis, 100 cells were randomly selected, and the fluorescent changes of the cells were measured between 2 seconds and 22 seconds after stimulation.

### Measurement of the Responses of hTRPA1- or hTRPV1-expressing Cells Using a Plate Reader-based Assay

The trypsinized cells were seeded at a density of 70,000 cells (for hTRPA1) or 60,000 cells (for hTRPV1) per well into 96-well black-wall CellBIND surface plates (Corning, Corning, NY, USA). After 17–23 hrs, the cells were washed with the assay buffer prior to loading with the calcium indicator dye of the FLIPR Calcium 4 Assay kit (Molecular Devices). The cells were incubated for 45 min at 27°C, and the measurements were obtained using FlexStation 3 (Molecular Devices). The changes in fluorescence (excitation at 485 nm, emission at 525 nm, and cutoff at 515 nm) were monitored at 2-sec intervals for 120 sec. An aliquot of 100 µl of the assay buffer containing the test compound was added 20 sec after beginning the experiment, and scanning was continued for an additional 100 sec. The final concentrations of AITC and artepillin C were 50 µM and 10 µM, respectively; the other compounds were used at 100 µM.

Dose-response curves were generated using the same method described above, and each curve set contained 8 data points. For the calculation of the half-maximal effective concentration (EC_50_) values, plots of the amplitudes versus the concentrations were prepared using Clampfit Version 9.2.0.09 (Molecular Devices). Nonlinear regression of the plot produced the function 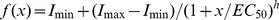, where 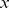 is the ligand concentration and 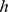 is the Hill coefficient, which was used to calculate the EC_50_ values for the ligand-receptor interactions. The response of each well was represented as ΔRFU (delta relative fluorescence unit), which was defined as follows: ΔRFU = (maximum fluorescence value) - (minimum fluorescence value). The responses were averaged from at least three wells receiving the same stimulus.

### Statistical Analysis

The data are presented as the mean ± SEM and were assessed for statistical significance using Tukey’s multiple comparison test. The equality of the variances was measured using the Levene’s test with commercial software for statistical analysis (Ekuseru-Toukei 2006, Social Survey Research Information Co., Ltd., Tokyo, Japan). The differences were considered to be significant when the *p* value was less than 0.05.
